# Oxidative damage and response to *Bacillus Calmette-Guérin* in bladder cancer cells expressing sialyltransferase ST3GAL1

**DOI:** 10.1186/s12885-018-4107-1

**Published:** 2018-02-17

**Authors:** Paulo F. Severino, Mariana Silva, Mylene Carrascal, Nadia Malagolini, Mariella Chiricolo, Giulia Venturi, Roberto Barbaro Forleo, Annalisa Astolfi, Mariangela Catera, Paula A. Videira, Fabio Dall’Olio

**Affiliations:** 1 Centro de Estudos de Doenças Crónicas, CEDOC, NOVA Medical School/Faculdade de Ciências Médicas, Universidade NOVA de Lisboa, Campo dos Mártires da Pátria, 130, 1169-056 Lisbon, Portugal; 2 UCIBIO, Departamento Ciências da Vida, Faculdade de Ciências e Tecnologia, Universidade NOVA de Lisboa, 2829-516 Caparica, Portugal; 3 Dipartimento di Medicina Specialistica, Diagnostica e Sperimentale, Sede di Patologia Generale, Università di Bologna, Via S. Giacomo 14, 40126 Bologna, Italy; 4 Centro Interdipartimentale Ricerche sul Cancro “Giorgio Prodi”, Università di Bologna, Bologna, Italy

**Keywords:** Bacillus *Calmette-Guérin*, Glycosylation, Sialyl T antigen, Sialyltransferase, Thomsen-Friedenreich antigen

## Abstract

**Background:**

Treatment with *Bacillus Calmette-Guérin* (BCG) is the gold standard adjuvant immunotherapy of non-muscle invasive bladder cancer (NMIBC), although it fails in one third of the patients. NMIBC expresses two tumor-associated *O*-linked carbohydrates: the disaccharide (Galβ1,3GalNAc) Thomsen-Friedenreich (T) antigen, and its sialylated counterpart (Siaα2,3Galβ1,3GalNAc) sialyl-T (sT), synthesized by sialyltransferase ST3GAL1, whose roles in BCG response are unknown.

**Methods:**

The human bladder cancer (BC) cell line HT1376 strongly expressing the T antigen, was retrovirally transduced with the *ST3GAL1* cDNA or with an empty vector, yielding the cell lines HT1376_sT_ and HT1376_T_, that express, respectively, either the sT or the T antigens. Cells were in vitro challenged with BCG. Whole gene expression was studied by microarray technology, cytokine secretion was measured by multiplex immune-beads assay. Human macrophages derived from blood monocytes were challenged with the secretome of BCG-challenged BC cells.

**Results:**

The secretome from BCG-challenged HT1376_sT_ cells induced a stronger macrophage secretion of IL-6, IL-1β, TNFα and IL-10 than that of HT1376_T_ cells. Transcriptomic analysis revealed that *ST3GAL1* overexpression and T/sT replacement modulated hundreds of genes. Several genes preserving genomic stability were down-regulated in HT1376_sT_ cells which, as a consequence, displayed increased sensitivity to oxidative damage. After BCG challenge, the transcriptome of HT1376_sT_ cells showed higher susceptibility to BCG modulation than that of HT1376_T_ cells.

**Conclusions:**

High *ST3GAL1* expression and T/sT replacement in BCG challenged-BC cancer cells induce a stronger macrophage response and alter the gene expression towards genomic instability, indicating a potential impact on BC biology and patient’s response to BCG.

**Electronic supplementary material:**

The online version of this article (10.1186/s12885-018-4107-1) contains supplementary material, which is available to authorized users.

## Background

The intravesical inoculation with the *Bacillus Calmette-Guérin* (BCG) is the most effective adjuvant therapy of non-muscle invasive bladder cancers (NMIBC) after transurethral resection. However, one third of the patients fail to respond and experience recurrence after treatment. The reasons why BCG therapy fail are still unclear, although it is well established that the anti-tumor activity of BCG depends on its ability to elicit an effective local immune response [1–3].

Glycosylation, one of the most frequent post-translational modification of proteins, undergoes profound changes in all types of cancer [4], including bladder cancer (BC) [5–8]. The aberrant expression of glycoconjugates is often caused by the deranged regulation of their biosynthetic enzymes: the glycosyltransferases [9]. The Thomsen-Friedenreich (T) antigen is a disaccharide (Galβ1,3GalNAc) *O*-linked to serine or threonine residues of glycoproteins (Additional file 1A), whose aberrant expression in cancer has been associated with malignancy [10–13] and used as a possible target for therapy [14–16]. Its sialylated counterpart, the sialyl-T (sT) (Siaα2,3Galβ1,3GalNAc-*O*-Ser/Thr) structure and its main biosynthetic enzyme, the sialyltransferase ST3GAL1, are also aberrantly expressed in a variety of cancers [17, 18] [reviewed in [19, 20]]. In BC, the expression of T/sT antigens is also aberrant and it influences invasion and immune recognition [21–23]. In a previous work [24], we have shown that the mRNA of *ST3GAL1* was overexpressed in NMIBC but not in muscle invasive BC or in benign bladder tumors and ST3GAL1 plays the major role in the sialylation of the T antigen in BC. The T antigen has been suggested as a useful marker of BCG response [23], even though the relationship between ST3GAL1/sT and BCG response has never been established.

In this study, we investigated the effects of the alternative expression of the T or sT antigens on the ability of BC cells to activate macrophages in response to BCG challenge and on the transcriptome of BC cells, utilizing the HT1376 cell line in which the T antigen was replaced by the sT antigen, by retroviral transduction with the *ST3GAL1* cDNA. This cell line was chosen because of its low ST3GAL1 expression and its high and homogenous reactivity with the T antigen-specific lectin PNA [24]. The gene expression and cytokine profiles of the cell lines expressing either the T or the sT antigens after BCG challenge and the ability of their secretome to stimulate cytokine release by macrophages was studied.

## Methods

### Generation of ST3GAL1-expressing cell lines

The HT1376 cell line was established from a primary invasive transitional cell cancer of the bladder [25]. Cells were grown in DMEM (4.5 g/L glucose, Sigma), containing 10% foetal calf serum (FCS, Sigma), 2 mM *L*-glutamine (Sigma) and 100 μg/mL penicillin/streptomycin (Sigma). HT1376 cells expressing *ST3GAL1* were generated by transduction with a retroviral vector obtained with the ViraPower Lentiviral Expression System (Invitrogen), according to manufacturer’s instructions. The cDNA of the whole coding region of human *ST3GAL1* was obtained by PCR amplification of the cDNA of the colon cancer cell line HT29 with the following primer pair: forward primer: 5’-CACCATGGTGACCCTGCGGAAGAGG-3′; reverse primer: 5’-TCATCTCCCCTTGAAGATCCGG-3′. Amplification was performed for 35 cycles of the following program: denaturation 94 °C 1 min; annealing 60 °C 1 min; extension 72 °C 1 min. The PCR product was gel isolated and cloned into the pLenti6/V5 Directional TOPO cloning vector (Invitrogen) which drives the expression of inserted genes under the control of the cytomegalovirus promoter. A negative control retroviral vector was prepared with an empty plasmid. After transduction with negative control- or *ST3GAL1*-expressing vectors, HT1376 cells were selected with 4 μg/mL of blasticidin. The replacement of the T antigen with the sT antigen was evaluated as loss of cell reactivity with the fluorescent labeled lectin from *Arachis hypogea* (peanut agglutinin, PNA), conjugated with fluorescein isothiocyanate (PNA-FITC). Although selected cells were mainly negative to PNA-FITC as detected by FACS analysis, a small population of PNA-FITC positive cells was still present. To obtain a population of cells homogeneously negative for the T antigen, about 100 *ST3GAL1*-transduced HT1376 cells were seeded in a 10 cm Petri dish and after one month, PNA-FITC negative colonies were selected and pooled. This polyclonal cell population homogeneously negative for T antigen expression is thereafter referred to as HT1376_sT_. The polyclonal cell population obtained after transduction with the negative control retroviral vector followed by blasticidin selection is referred to as HT1376_T_.

### Flow cytometry

Cells were incubated with PNA-FITC for 30 min at 4 °C in the dark, washed and analyzed by flow cytometry. Sialidase treatment was performed with 20 mU of *Clostridium perfringens* sialidase (Roche Diagnostics), for 90 min at 37 °C.

### Real time RT-PCR

Total RNA was isolated using the GenElute Mammalian Total RNA Purification kit and DNase treatment (Sigma), according to the manufacturer’s instructions. One microgram of total RNA was reverse transcribed, using the random-primers based High Capacity cDNA Archive Kit (Applied Biosystems). The expression level of *ST3GAL1* (Hs00161688_m1; NM_173344.2 and NM_003033.3) was evaluated with the TaqMan assay system in a 7500 Fast Real-Time PCR System (Applied Biosystems) using the TaqMan Universal PCR Master Mix Fast, as previously described [24, 26, 27]. The efficiency of the amplification reaction for each primer-probe was above 95% (as determined by the manufacturer). Normalized mRNA expression was computed as the number of mRNA molecules of the gene of interest *per* 1000 mRNA molecules of the endogenous control β-actin gene, calculated using the 2^-ΔCT^×1000 formula [28].

### Sialyltransferase activity assay

Cell pellets were homogenized in water and the protein concentration of the homogenates was determined by the Lowry method. The activity of ST3GAL1 was measured in the homogenates in the range of time and substrate concentration linearity in a 25 μL volume containing: 50 mM of 2-(*N*-morpholino)ethanesulphonic acid (MES) buffer pH 6.5, 0.5% Triton X-100, 23.5 μg of Galβ1,3GalNAcα1-*O*-benzyl (benzyl-T; Sigma) as acceptor substrate, 15 μM (640 Bq) of CMP-[^14^C]Sia (Amersham) and 50 μg of homogenate proteins. Reactions were incubated at 37 °C, for 2 h and the products were then isolated by hydrophobic chromatography in SepPak C18 Classic Cartridge (Waters). The columns were washed with water and eluted with 1 mL acetonitrile, which was counted in a liquid scintillation counter. The incorporation on endogenous substrates, in the absence of the acceptor substrate, was subtracted.

### BCG challenge of HT1376 cells

Commercial Connaught BCG (ImmuCyst, Sanofi Pasteur SA, France) was suspended in PBS containing 0.05% Tween 80 and stored at − 80 °C. Before each assay, BCG aggregates were discarded by centrifugation (300 × g for 5 min). To assess BCG internalization, bacteria were stained with 2 μg/mL of 5-(and-6-)(((4-chloromethyl)benzoyl)amino)tetramethylrhodamine (CMTR, Invitrogen) for 2 h in culture medium, incubated with HT1376_T_ or HT1376_sT_ cells in a 1:10 cell/bacteria ratio for 2 h at 37 °C and analyzed by flow cytometry. To assess cytokine secretion, HT1376_T_ or HT1376_sT_ cells were challenged with unstained BCG for 2 h at 37 °C, the medium was removed and the cells were washed twice with PBS and incubated with fresh medium for 16 h. Conditioned media were used for cytokine analysis and to challenge macrophages, while cell pellets were used for RNA extraction and transcriptomic analysis.

### Determination of cytokine concentration

The concentration of cytokines IL-1β, IL-2, IL-4, IL-6, IL-8, IL-10, IL-12, IL-17, IFN-γ and TNF-α was measured in a 96-well strip plate from a commercial MIBA kit (Bio-Rad), as recommended by manufacturer’s instructions. Fluorescence was read in a Luminex 100 Bio-Plex Liquid Array Multiplexing System reader (Bio-Rad) and the data analyzed with the Bio-Plex Manager v5 software (Bio-Rad).

### Macrophage preparation and stimulation

Mononuclear cells were isolated by Ficoll-Hypaque density gradient centrifugation (GE Healthcare) from the peripheral blood of healthy blood-donors. For this use, no study approval was necessary. The only authorization required was that obtained from the Blood Collection Service of the Pizzardi Hospital in Bologna, Italy, which keeps the rights on donors’ blood samples. Macrophages were obtained by differentiation of monocytes by culture in RPMI 1640 (Sigma) medium supplemented with 20% FCS, 2 mM L-glutamine and 100 μg/mL penicillin/streptomycin. After 7 days, monocyte-derived macrophages were detached with a cell scraper and dispensed in 24 well plates at a cell density corresponding approximately to 50% of confluence. One day later, macrophages were incubated with standard unconditioned culture medium or with the media conditioned by HT1376_T_ or HT1376_sT_ cells either BCG-challenged (as described above) or mock challenged. After 2 h, the conditioned media were replaced by fresh medium, which was collected 24 h later and stored at − 80 °C for the detection of cytokines secreted by macrophages.

### H_2_O_2_ treatment

Cells in exponential growth phase were incubated in serum-free medium containing 5 mM H_2_O_2_ for 1 h. The medium was then replaced with fresh complete medium and the cells were harvested and analyzed 24 h later. Mock-treated cells were incubated as above without H_2_O_2_. The cytotoxic effect of H_2_O_2_ was determined by counting the number of cells in six replicas seeded in 6-wells plates. Representative fields were photographed with an inverted phase contrast microscope.

### Whole transcriptome analysis by expression microarray

Total RNA was isolated by the guanidinium thiocyanate-method [29] and converted to labelled single strand cDNA (ssDNA) by the commercial Whole Transcript Expression kit (Ambion), according to the manufacturer’s instructions. Labelled ssDNA fragments were hybridized in a Human Transcriptome Array 2.0 overnight. After staining with phycoerythrin-streptavidin, fluorescence was read in a GeneChip Scanner 3000 7G (Affymetrix). After statistical analysis (see below), array data were functionally analyzed by the ArrayStar v2.0 software (DNASTAR) and through a literature search of the biological roles of modulated genes. Gene nomenclature followed the *HUGO Gene Nomenclature Committee* rules (https://www.genenames.org/) in italic uppercase letters. With exception of cytokines, proteins had the same name as the gene, represented in regular uppercase.

### Statistical methods

Microarray raw data were background-subtracted, normalized and summarized with the robust multi-array average (RMA) algorithm implemented in the Affy package of Bioconductor (www.bioconductor.org), which utilizes R software. Differentially expressed genes between query and control assay were selected by application of the two tail ANOVA, followed by the Benjamini-Hochberg false discovery rate test with a *p* or *q* ≤ 0.05 cut-off and by the log_2_ expression ratio, considering only variations ≥0.5. MIBA data were analyzed by ANOVA, followed by Tukey multiple comparison test. H_2_O_2_ toxicity data were analyzed by the Student’s *t* test. The software used was Graphpad Prism, version 7.0.

## Results

### ST3GAL1-expression leads to replacement of T with sT antigen in HT1376 cells

The mock-transduced cell line HT1376_T_, like the wild type HT1376, expressed high and homogeneous T expression, while the *ST3GAL1*-transduced cell line HT1376_sT_ displayed an homogeneous low PNA reactivity which could be reverted to high reactivity after sialidase treatment (Additional file 1B). Both the ST3GAL1 activity (Additional file 1C) and the ST3GAL1 mRNA measured by real time PCR (Additional file 1D) were very high in HT1376_sT_ although almost undetectable in HT1376_T_. The level of ST3GAL1 activity and PNA reactivity reached by HT1376_sT_ cells was similar to that displayed by the wild type BC cell line 5637 strongly expressing ST3GAL1 (data not shown [24]). This is consistent with the notion that, in BC cells, ST3GAL1-mediated α2,3 sialylation of galactose (Additional file 1A) masks the T antigen [24].

### IL-6 and IL-8 secretion by HT1376_T_ and HT1376_sT_ cells

**Fig. 1:**
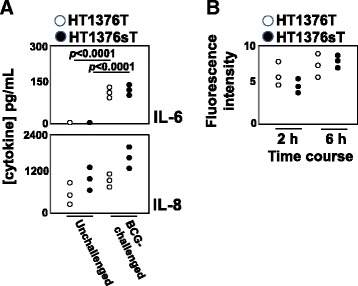
BCG-stimulation of HT1376 cells. **a**: Secretion of IL-6 and IL-8. HT1376_T_, (white circles) and HT1376_sT_ (black circles) cells were challenged with BCG for 2 h, then they were washed and cultured in medium for 16 h. Cytokine concentration was determined as described in Materials and Methods section. Data of three independent experiments are reported. Statistical analysis is reported in Additional file 2. **b**: Time course of BCG uptake. HT1376T (white circles) and HT1376sT (black circles) cells were challenged with CMTMR-labelled BCG for 2 and 6 h, as described in the Methods section. BCG internalization was estimated as mean fluorescence intensity of the cells, measured by flow cytometry Data of three independent experiments are reported

To assess the effect of *ST3GAL1* overexpression and the consequent replacement of the T with the sT antigen on cytokine production following BCG stimulation, we challenged the two HT1376 cell lines with BCG. Among the several cytokines tested (IL-1β, IL-2, IL-4, IL-6, IL-8, IL-10, IL-12, IL-17, IFN-γ and TNF-α), only IL-8 was detectable in media conditioned by unchallenged HT1376_T_ or HT1376_sT_ cells. After BCG challenge, IL-6 became detectable in both HT1376_sT_ and HT1376_T_ cells, while the IL-8 secretion showed a clear tendency to up-regulation after BCG challenge, especially in HT1376_T_ cells (Fig. 1a). The *p* values are reported in Additional file 2.

To establish whether the different IL-8 production by HT1376_sT_ after BCG challenge was due to a higher BCG uptake by these cells, we measured BCG internalization by the two HT1376 cell populations by flow cytometry. As shown in Fig. 1b, there were no significant differences in the uptake of fluorescent BCG by HT1376_T_ and HT1376_sT_ cells, ruling out the possibility that differences in cytokine production by BCG-stimulated HT1376_T_ and HT1376_sT_ was dependent on different BCG internalization rate.

### HT1376_sT_ cells induce a stronger macrophage secretory response after BCG stimuli

**Fig. 2:**
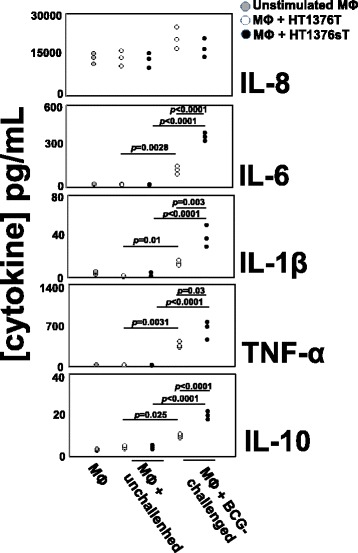
Cytokine secretion by human macrophages treated with conditioned media of BC cell lines. Cytokine secretion was measured in culture media conditioned by unstimulated macrophages (MΦ, grey circles) or stimulated with conditioned media from BCG-challenged or unchallenged BC cell lines: HT1376_T_, (white circles); HT1376_sT_ (black circles). Cells were challenged with BCG for 2 h, then they were washed and allowed to condition the culture medium for 16 h. The conditioned culture media of BCG-challenged or unchallenged cells was used to stimulate human monocyte-derived macrophages for 24 h. MØ: cytokines released by unstimulated macrophages; MØ + unchallenged: cytokines released by macrophages stimulated with the conditioned medium of unchallenged HT1376 cells; MØ + BCG-challenged: cytokines released from macrophages stimulated with the conditioned medium of BCG-challenged cells. Data of three independent experiments are reported. Statistical analysis is reported in Additional file 2

To investigate the role played by ST3GAL1 expression and the consequent T/sT replacement on the stimulation of innate immunity by BCG-challenged BC cells, the secretion of cytokines by human macrophages stimulated with the secretome of BCG-challenged BC cells was measured. Unstimulated macrophages secreted high levels of IL-8 and very low levels of TNF-α, IL-6, IL-1β and IL-10. This pattern of secretion was not significantly changed by stimulation with the secretome of BCG-unchallenged HT1376_T_ or HT1376_sT_ cells (Fig. 2). In contrast, the secretome from both BCG-challenged cell lines, in particular that from HT1376_sT_, significantly increased the secretion of IL-6, IL-1β, TNF-α and IL-10 whereas IL-8 secretion was hardly affected by the secretome of BCG-challenged cells, regardless of their ST3GAL1 expression (Fig. 2). It should be noted that the level of secretion of IL-8 and IL-6 by macrophages was about 10 and 3-fold higher, respectively, than the level of either in HT1376 cells (Fig. 1), ruling out the possibility of a significant contamination of cytokines secreted by macrophages with those secreted by HT1376 cells. The cytokines IL-2, IL-4, IL-12 and IL-17 were not secreted by macrophages in any of the tested conditions (data not shown). The *p* and *q* values are reported in Additional file 2.

### HT1376_sT_ cells display down-regulation of genes preserving genomic stability

**Fig. 3:**
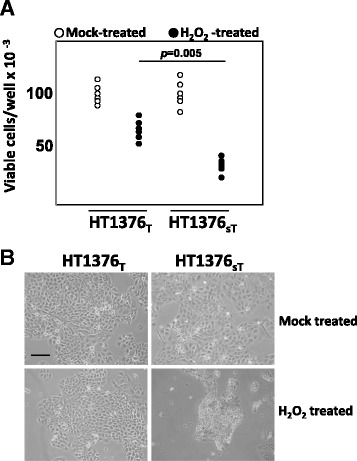
Cytotoxic effect of H_2_O_2_ on HT1376_T_ and HT1376_sT_ cells. **a**: cells were treated with 5 mM H_2_O_2_ or mock-treated as described in Methods and counted. Data of six experiments are reported. The HT1376_sT_ cells are more susceptible to the toxic effect of H_2_O_2_ (**p = 0.005 according to Student’s *t* test). **b**: phase contrast micrograph of representative fields of the two cell populations treated as described above, showing the stronger cytotoxic effects of H_2_O_2_ on HT1376_sT_. Scale bar: 100 μm

The impact of ST3GAL1 overexpression and of T replacement by sT on the global transcriptional activity of HT1376 cells was evaluated by expression microarray technology. Of the 254 genes, which displayed ST3GAL1-dependent modulation by at least a log_2_ expression ratio ≥ 1.0 (meaning a fold change of at least 2) with *p* or *q* values ≤0.05, 106 were up-regulated and 148 were down-regulated. The complete list of genes showing significant modulation (changes equal or higher than 2-fold) in HT1376_sT_ vs. HT1376_T_ is reported in Additional file 3. At least 29 genes putatively regulating malignancy because of their involvement in the control of apoptosis, cell growth, angiogenesis, inflammation or proteolysis, were differentially expressed in HT1376_sT_ cells, compared with HT1376_T_ cells (Additional file 4). A search in the literature revealed that 18 genes were modulated towards increased malignancy and 14 towards decreased malignancy (Additional file 4). Remarkably, among the 18 genes modulated towards increased malignancy in HT1376_sT_ cells, 11 down-regulated genes can be collectively referred to as “caretaker genes” because of their involvement in DNA repair and mitotic fidelity (Additional file 4). Moreover, if threshold was lowered to include genes down-regulated above 1.3-fold, the number of down-regulated caretaker genes rose to 34 (Table 1). As a functional validation of gene expression data, cells were exposed to the genotoxic effects of the oxidizing agent H_2_O_2_. As expected for a cell line with impaired DNA repair mechanisms, H_2_O_2_ -treatment resulted in a reduced viability in HT1376_sT_ than in HT1376_T_ cells (Fig. 3a and b).

**Table 1:**
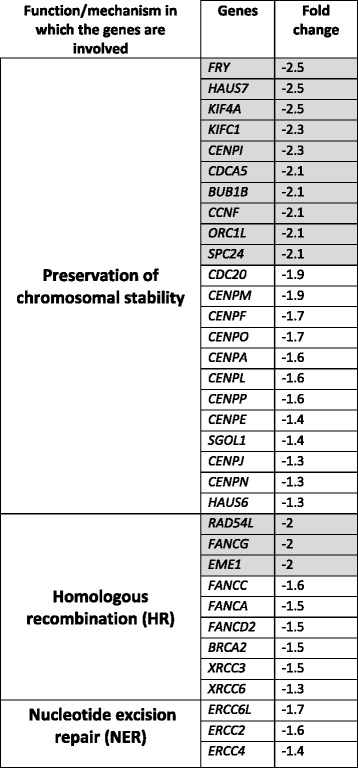
Genes involved in maintaining chromosomal stability and/or DNA repair showing down-regulation in HT1376_sT_ cells

Genes showing a fold change ≥ 2 are boxed in gray

### HT1376_sT_ cells display increased transcriptomic changes after BCG challenging

To investigate the effect of a differential T/sT expression on BCG challenging, the global transcriptional activity was also compared in BCG-challenged HT1376_T_ and HT1376_sT_ cell lines. While in HT1376_sT_ cells a group of 38 genes showed significant modulation by BCG (*p* and *q* values ≤0.05) with a log_2_ expression ratio ≥ 0.5 (fold change at least 1.4) (Additional information 5), the tendency to modulation showed by a few genes in HT1376_T_ cells never reached statistical significance (data not shown). Modulated genes in HT1376_sT_ cells fell into several broad functional categories (Additional information 5). In particular, a set of genes related to “aminoacid and protein biosynthesis” were prevalently down-regulated in BCG-challenged HT1376_sT_ cells (Additional file 5). This functional category includes genes involved in the biosynthesis and transport of aminoacids, attachment of aminoacids to tRNAs and regulation of translation. Other categories of modulated genes include “cell growth” and “inflammatory and immune response” (Additional file 5)*.*

## Discussion

The overexpression of ST3GAL1 has been widely studied in cellular models of breast cancer and found to be responsible for increased malignancy [30–32]. In contrast, very little is known on the role of this enzyme in BC biology. In this work, we have performed an exhaustive analysis of a BC experimental system in which through the overexpression of ST3GAL1, the constitutively expressed T antigen was replaced by its sialylated counterpart, the sT carbohydrate structure. Both structures are biologically active, being receptors either for galectins or siglecs, as well as for sugar binding receptors of microorganisms [33]. Thus, the phenotypic changes we report after ST3GAL1 overexpression could be attributable either to the lack of T antigen or expression of sT or both. The modulation of the immune response, in particular, the secretion of cytokines, is a well known functional consequence of BCG interaction with BC cells [1, 34, 35]. Our study confirms that BCG induces IL-6 and IL-8 secretion by BC cells and shows a tendency to higher IL-8 secretion by BCG-stimulated cells expressing sT. In BC patients, plasma [36] and tissue [37] IL-6 levels are elevated and associated with tumor progression and poor prognosis. Previous studies have indicated that BCG stimulates IL-6 production in urine of patients and BC cell lines [38], inducing non apoptotic cell death [39]. Increased urinary IL-8 level also correlates with progression in BC patients [40], although a high urinary IL-8 level after BCG instillation is a prognostic factor of successful outcome [41, 42]. Our data indicate that after BCG challenge, only the secretion of IL-6 and, to a lesser extent, IL-8 were stimulated while the other cytokines remained undetectable. This is consistent with clinical observations reporting that a few cytokines, including IL-6 and IL-8, were detectable in urine after a first intravesical BCG administration, while other cytokines required multiple BCG instillations [43].

After BCG challenge, the secretome of BC cells expressing the sT structure showed higher capacity to stimulate cytokine secretion by macrophages, further supporting the notion that the sT antigen potentiates the BC response to BCG.

An inflammatory environment can either promote or inhibit tumor progression, consistent with the notion that inflammation is a double edge sword which can both eradicate the tumor but also fuel its growth. Macrophages are differentiated either as pro-inflammatory M1 or anti-inflammatory M2 phenotypes, even if in many cases they are in an intermediate condition. While a variable capacity to secrete IL-6, IL-1β and TNF-α is shared by differentially polarized macrophages, an IL-12 low/IL-10 high phenotype is the hallmark of M2 macrophages [44], which are known to promote tumor growth [45]. The macrophages used in this study secreted IL-6, IL-8, IL-1β and TNF-α but no IL-12 and little IL-10 and are probably representative of an intermediate condition between the two extreme phenotypes. The nature of the macrophages associated with BC is indeed variable as indicated by the different level of expression of the M2-specific marker CD163 among patients [46]. Interestingly, the predominance of M2 macrophages is associated with higher stage and grade [46] and with a worse response to BCG [47].

The most prominent transcriptomic change we observed because of *ST3GAL1* expression and the consequent replacement of the T with the sT antigen in HT1376 cells was the decreased expression of several genes involved in different mechanisms of DNA repair and in the accuracy of chromosomal segregation. This resulted in increased sensitivity of HT1376_sT_ cells to the cytotoxic effect of H_2_O_2._ This is of interest if one considers that the generation of reactive oxygen species by BCG is a crucial mechanism of BCG-induced damage to BC cells [48]. Interestingly, also in glioma cells, ST3GAL1 expression resulted in transcriptomic changes affecting malignancy and the cell cycle [49], supporting the notion that a carbohydrate structure on the cell surface can generate an “outside in” flow of information modulating gene expression [20].

BCG challenge resulted in a deeper modulation of the transcriptome in HT1376_sT_ than in HT1376T, suggesting that the replacement of the T with the sT antigen on the cell surface changes the development of the genetic program triggered by BCG contact.

Even though genes involved in inflammatory and immune response were found to be modulated by BCG challenge in HT1376sT cells, little or no changes were observed in genes encoding cytokines, including those whose expression was stimulated by BCG-challenge. Possible discrepancies between gene and protein expression can be explained considering that multiple mechanisms operating at postranscriptional and postranslational levels (including non-coding RNAs, translation regulatory mechanisms, proteasomal activity) regulate protein expression. In addition, as shown for IL-1β, cytokine secretion can be regulated by the release of preformed molecules from intracellular stores, rather than at the level of gene transcription [50]. The soluble factors responsible for the stimulation of cytokine secretion by macrophages are conceivably a very complex cocktail of bioactive compounds of protein and non-protein nature, including biologically active molecules (e.g. prostaglandins, glycosaminoglycans) which are not primary gene products but products of multiple enzymatic reactions. For these reasons, the nature of the molecules secreted by BC cells responsible for the effect on macrophages may not be directly related to the gene expression profile.

## Conclusions

In conclusion, our data show that ST3GAL1 expression and the consequent replacement of the T by the sT antigen in BC cells induce transcriptomic changes with a putative impact on multiple cellular functions associated with increased malignancy and susceptibility to oxidative damage and strengthens the inflammatory response of macrophages. This indicates that ST3GAL1 and T/sT carbohydrate structures can be factors with multiple clinical implications in BC.

## Additional files

Additional file 1: **Figure S1.** *ST3GAL1*-overexpression in HT1376 cells (PPTX 130 kb)

Additional file 2: *p* values calculated with ANOVA, followed by Tukey multiple comparison test for data reported in Fig. 1 and Fig. 2 (PPTX 84 kb)

Additional file 3: GENES MODULATED IN HT1376sT CELLS AS COMPARED TO HT1376T CELLS. (XLSX 47 kb)

Additional file 4: CANCER-ASSOCIATED GENES MODULATED IN HT1376sT CELLS AS COMPARED TO HT1376T CELLS. (XLS 43 kb)

Additional file 5: GENES MODULATED BY BCG IN HT1376_sT_ (XLS 49 kb)

## Abbreviations

BC: bladder cancer
BCG: *Bacillus Calmette-Guérin*
CMTMR: 5-(and-6)-(((4-chloromethyl)benzoyl)amino)tetramethylrhodamine
DMEM: Dulbecco’s modified Eagle medium
FCS: Foetal calf serum
FITC: Fluorescein isothiocyanate
Gal: Galactose
GalNAc: N-acetylgalactosamine
GlcNAc: N-acetylglucosamine
MES: (*N*-morpholino)ethanesulphonic acid
MIBA: Multiplex immune-beads assay
NMIBC: Non-muscle invasive bladder cancer
PBS: Phosphate-buffered saline
PNA: Peanut (*Arachis hypogea*) agglutinin
RPMI: Roswell Park Memorial Institute
Sia: sialic acid

## References

[1] Bevers RF, Kurth KH, Schamhart DH (2004). Role of urothelial cells in BCG immunotherapy for superficial bladder cancer. Br J Cancer.

[2] Videira PA, Calais FM, Correia M, Ligeiro D, Crespo HJ, Calais F, Trindade H (2009). Efficacy of bacille Calmette-Guerin immunotherapy predicted by expression of antigen-presenting molecules and chemokines. Urology.

[3] Carretero R, Cabrera T, Gil H, Saenz-Lopez P, Maleno I, Aptsiauri N, Cozar JM, Garrido F (2011). Bacillus Calmette-Guerin immunotherapy of bladder cancer induces selection of human leukocyte antigen class I-deficient tumor cells. Int J Cancer.

[4] Dall'Olio F, Malagolini N, Trinchera M, Chiricolo M (2012). Mechanisms of cancer-associated glycosylation changes. Front Biosci.

[5] Ohyama C (2008). Glycosylation in bladder cancer. Int J Clin Oncol.

[6] Yang G, Tan Z, Lu W, Guo J, Yu H, Yu J, Sun C, Qi X, Li Z, Guan F (2015). Quantitative Glycome analysis of N-glycan patterns in bladder cancer vs normal bladder cells using an integrated strategy. J Proteome Res.

[7] Ferreira JA, Videira PA, Lima L, Pereira S, Silva M, Carrascal M, Severino PF, Fernandes E, Almeida A, Costa C (2013). Overexpression of tumour-associated carbohydrate antigen sialyl-Tn in advanced bladder tumours. Mol Oncol.

[8] Lima L, Severino PF, Silva M, Miranda A, Tavares A, Pereira S, Fernandes E, Cruz R, Amaro T, Reis CA (2013). Response of high-risk of recurrence/progression bladder tumours expressing sialyl-Tn and sialyl-6-T to BCG immunotherapy. Br J Cancer.

[9] Brockhausen I (1999). Pathways of O-glycan biosynthesis in cancer cells. Biochim Biophys Acta.

[10] Glinsky VV, Glinsky GV, Rittenhouse-Olson K, Huflejt ME, Glinskii OV, Deutscher SL, Quinn TP (2001). The role of Thomsen-Friedenreich antigen in adhesion of human breast and prostate cancer cells to the endothelium. Cancer Res.

[11] Zhao Q, Barclay M, Hilkens J, Guo X, Barrow H, Rhodes JM, Yu LG (2010). Interaction between circulating galectin-3 and cancer-associated MUC1 enhances tumour cell homotypic aggregation and prevents anoikis. Mol Cancer.

[12] Glinsky VV, Huflejt ME, Glinsky GV, Deutscher SL, Quinn TP (2000). Effects of Thomsen-Friedenreich antigen-specific peptide P-30 on β-galactoside-mediated homotypic aggregation and adhesion to the endothelium of MDA-MB-435 human breast carcinoma cells. Cancer Res.

[13] Engelstaedter V, Fluegel B, Kunze S, Mayr D, Friese K, Jeschke U, Bergauer F (2012). Expression of the carbohydrate tumour marker Sialyl Lewis a, Sialyl Lewis X, Lewis Y and Thomsen-Friedenreich antigen in normal squamous epithelium of the uterine cervix, cervical dysplasia and cervical cancer. Histol Histopathol.

[14] Almogren A, Abdullah J, Ghapure K, Ferguson K, Glinsky VV, Rittenhouse-Olson K (2012). Anti-Thomsen-Friedenreich-ag (anti-TF-ag) potential for cancer therapy. Front Biosci (Schol Ed).

[15] Ferguson K, Yadav A, Morey S, Abdullah J, Hrysenko G, Eng JY, Sajjad M, Koury S, Rittenhouse-Olson K (2014). Preclinical studies with JAA-F11 anti-Thomsen-Friedenreich monoclonal antibody for human breast cancer. Future Oncol.

[16] Heimburg J, Yan J, Morey S, Glinskii OV, Huxley VH, Wild L, Klick R, Roy R, Glinsky VV, Rittenhouse-Olson K (2006). Inhibition of spontaneous breast cancer metastasis by anti-Thomsen-Friedenreich antigen monoclonal antibody JAA-F11. Neoplasia.

[17] Brockhausen I, Yang JM, Burchell J, Whitehouse C, Taylor-Papadimitriou J (1995). Mechanisms underlying aberrant glycosylation of MUC1 mucin in breast cancer cells. Eur J Biochem.

[18] Whitehouse C, Burchell J, Gschmeissner S, Brockhausen I, Lloyd KO, Taylor-Papadimitriou J (1997). A transfected sialyltransferase that is elevated in breast cancer and localizes to the medial/trans-Golgi apparatus inhibits the development of core-2-based O-glycans. J Cell Biol.

[19] Dall'Olio F, Chiricolo M (2001). Sialyltransferases in cancer. Glycoconj J.

[20] Dall'Olio F, Malagolini N, Trinchera M, Chiricolo M (2014). Sialosignaling: Sialyltransferases as engines of self-fueling loops in cancer progression. Biochim Biophys Acta.

[21] Langkilde NC, Wolf H, Clausen H, Kjeldsen T, Orntoft TF (1992). Nuclear volume and expression of T-antigen, sialosyl-Tn-antigen, and Tn- antigen in carcinoma of the human bladder. Relation to tumor recurrence and progression. Cancer.

[22] Langkilde NC (1995). T-Antigens in primary non-invasive and superficially invasive human urinary bladder tumors: the correlation to tumor recurrence and tumor progression. A mini-review. Scand J Urol Nephrol Suppl.

[23] Dow JA, di Sant'Agnese PA, Cockett AT (1989). Expression of blood group precursor T antigen as a prognostic marker for human bladder cancer treated by bacillus Calmette-Guerin and interleukin-2. J Urol.

[24] Videira PA, Correia M, Malagolini N, Crespo HJ, Ligeiro D, Calais FM, Trindade H, Dall'Olio F (2009). ST3Gal.I sialyltransferase relevance in bladder cancer tissues and cell lines. BMC Cancer.

[25] Rasheed S, Gardner MB, Rongey RW, Nelson-Rees WA, Arnstein P (1977). Human bladder carcinoma: characterization of two new tumor cell lines and search for tumor viruses. J Natl Cancer Inst.

[26] Carrascal MA, Severino PF, Guadalupe CM, Silva M, Ferreira JA, Calais F, Quinto H, Pen C, Ligeiro D, Santos LL (2014). Sialyl Tn-expressing bladder cancer cells induce a tolerogenic phenotype in innate and adaptive immune cells. Mol Oncol.

[27] Crespo HJ, Cabral MG, Teixeira AV, Lau JT, Trindade H, Videira PA (2009). Effect of sialic acid loss on dendritic cell maturation. Immunology.

[28] Meijerink J, Mandigers C, van de Locht L, Tonnissen E, Goodsaid F, Raemaekers J (2001). A novel method to compensate for different amplification efficiencies between patient DNA samples in quantitative real-time PCR. J Mol Diagn.

[29] Chomczynski P, Sacchi N (1987). Single-step method of RNA isolation by acid guanidinium thiocyanate- phenol-chloroform extraction. Anal Biochem.

[30] Picco G, Julien S, Brockhausen I, Beatson R, Antonopoulos A, Haslam S, Mandel U, Dell A, Pinder S, Taylor-Papadimitriou J (2010). Over-expression of ST3Gal-I promotes mammary tumorigenesis. Glycobiology.

[31] Sproviero D, Julien S, Burford B, Taylor-Papadimitriou J, Burchell JM (2012). Cyclooxygenase-2 enzyme induces the expression of the α-2,3-Sialyltransferase-3 (ST3Gal-I) in breast cancer. J Biol Chem.

[32] Marcos NT, Cruz A, Silva F, Almeida R, David L, Mandel U, Clausen H, Mensdorff-Pouilly S, Reis CA (2003). Polypeptide GalNAc-transferases, ST6GalNAc-transferase I, and ST3Gal- transferase I expression in gastric carcinoma cell lines. J Histochem Cytochem.

[33] Burin d, Roziers N, Chadebech P, Bodivit G, Guinchard E, Bruneel A, Dupre T, Chevret L, Jugie M, Gallon P, Bierling P (2015). Red blood cell Thomsen-Friedenreich antigen expression and galectin-3 plasma concentrations in Streptococcus Pneumoniae-associated hemolytic uremic syndrome and hemolytic anemia. Transfusion.

[34] Bevers RF, de Boer EC, Kurth KH, Schamhart DH (1998). BCG-induced interleukin-6 upregulation and BCG internalization in well and poorly differentiated human bladder cancer cell lines. Eur Cytokine Netw.

[35] Chen FH, Crist SA, Zhang GJ, Iwamoto Y, See WA (2002). Interleukin-6 production by human bladder tumor cell lines is up-regulated by bacillus Calmette-Guerin through nuclear factor-kappaB and Ap-1 via an immediate early pathway. J Urol.

[36] Andrews B, Shariat SF, Kim JH, Wheeler TM, Slawin KM, Lerner SP (2002). Preoperative plasma levels of interleukin-6 and its soluble receptor predict disease recurrence and survival of patients with bladder cancer. J Urol.

[37] Chen MF, Lin PY, Wu CF, Chen WC, Wu CT (2013). IL-6 expression regulates tumorigenicity and correlates with prognosis in bladder cancer. PLoS One.

[38] Esuvaranathan K, Alexandroff AB, McIntyre M, Jackson AM, Prescott S, Chisholm GD, James K (1995). Interleukin-6 production by bladder tumors is upregulated by BCG immunotherapy. J Urol.

[39] Chen F, Zhang G, Cao Y, Hessner MJ, See WA (2009). MB49 murine urothelial carcinoma: molecular and phenotypic comparison to human cell lines as a model of the direct tumor response to bacillus Calmette-Guerin. J Urol.

[40] Sheryka E, Wheeler MA, Hausladen DA, Weiss RM (2003). Urinary interleukin-8 levels are elevated in subjects with transitional cell carcinoma. Urology.

[41] Sagnak L, Ersoy H, Ozok U, Senturk B, Ercil H, Bahar G, Ozturk E (2009). Predictive value of urinary interleukin-8 cutoff point for recurrences after transurethral resection plus induction bacillus Calmette-Guerin treatment in non-muscle-invasive bladder tumors. Clin Genitourin Cancer.

[42] Kumar A, Dubey D, Bansal P, Mandhani A, Naik S (2002). Urinary interleukin-8 predicts the response of standard and low dose intravesical bacillus Calmette-Guerin (modified Danish 1331 strain) for superficial bladder cancer. J Urol.

[43] Jackson AM, Alexandroff AB, Kelly RW, Skibinska A, Esuvaranathan K, Prescott S, Chisholm GD, James K (1995). Changes in urinary cytokines and soluble intercellular adhesion molecule-1 (ICAM-1) in bladder cancer patients after bacillus Calmette-Guerin (BCG) immunotherapy. Clin Exp Immunol.

[44] Balkwill F, Charles KA, Mantovani A (2005). Smoldering and polarized inflammation in the initiation and promotion of malignant disease. Cancer Cell.

[45] Mantovani A, Allavena P, Sica A, Balkwill F (2008). Cancer-related inflammation. Nature.

[46] Takeuchi H, Tanaka M, Tanaka A, Tsunemi A, Yamamoto H (2016). Predominance of M2-polarized macrophages in bladder cancer affects angiogenesis, tumor grade and invasiveness. Oncol Lett.

[47] Lima L, Oliveira D, Tavares A, Amaro T, Cruz R, Oliveira MJ, Ferreira JA, Santos L (2014). The predominance of M2-polarized macrophages in the stroma of low-hypoxic bladder tumors is associated with BCG immunotherapy failure. Urol Oncol.

[48] Shah G, Zielonka J, Chen F, Zhang G, Cao Y, Kalyanaraman B, See W (2014). H2O2 generation by bacillus Calmette-Guerin induces the cellular oxidative stress response required for bacillus Calmette-Guerin direct effects on urothelial carcinoma biology. J Urol.

[49] Chong YK, Sandanaraj E, Koh LW, Thangaveloo M, Tan MS, Koh GR, Toh TB, Lim GG, Holbrook JD, Kon OL, et al. ST3GAL1-associated transcriptomic program in glioblastoma tumor growth, invasion, and prognosis. J Natl Cancer Inst. 2016;108(2) 10.1093/jnci/djv326 PMC4755447 26547933

[50] Stanley AC, Lacy P (2010). Pathways for cytokine secretion. Physiology (Bethesda).

