# Supplementary material for: Oxidative damage and response to Bacillus Calmette-Guérin in bladder cancer cells expressing sialyltransferase ST3GAL1
Source: BMC Cancer. 2018 Feb 17;18:198. doi: 10.1186/s12885-018-4107-1 (PMC5816560; doi:10.1186/s12885-018-4107-1)
Supplement: Supplementary file 1 — Figure S1. ST3GAL1-overexpression in HT1376 cells (PPTX 130 kb) [file 12885_2018_4107_MOESM1_ESM.pptx]

## Slide 1
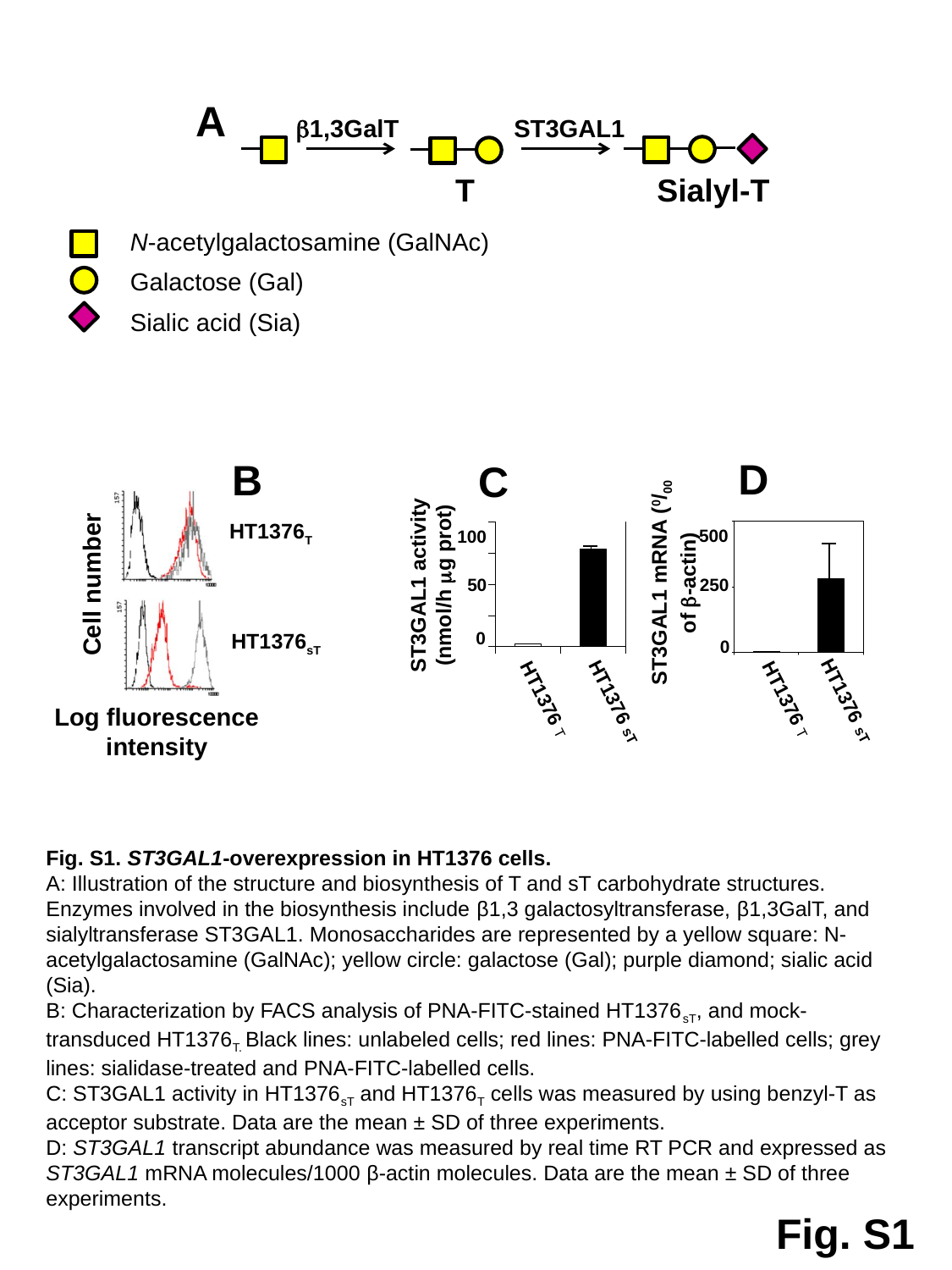

A
b1,3GalT
ST3GAL1
T
Sialyl-T
N-acetylgalactosamine (GalNAc)
Galactose (Gal)
Sialic acid (Sia)
D
B
C
HT1376T
### Chart
| Category | Activity |
|---|---|
| NC | 2.0 |
| ST3GAL1 | 78.0 |50
0
500
100
ST3GAL1 mRNA (0/00 of b-actin)
ST3GAL1 activity (nmol/h mg prot)
Cell number
250
HT1376sT
0
HT1376 T
HT1376 T
HT1376 sT
HT1376 sT
Log fluorescence intensity
Fig. S1. ST3GAL1-overexpression in HT1376 cells.
A: Illustration of the structure and biosynthesis of T and sT carbohydrate structures. Enzymes involved in the biosynthesis include β1,3 galactosyltransferase, β1,3GalT, and sialyltransferase ST3GAL1. Monosaccharides are represented by a yellow square: N-acetylgalactosamine (GalNAc); yellow circle: galactose (Gal); purple diamond; sialic acid (Sia).
B: Characterization by FACS analysis of PNA-FITC-stained HT1376sT, and mock-transduced HT1376T. Black lines: unlabeled cells; red lines: PNA-FITC-labelled cells; grey lines: sialidase-treated and PNA-FITC-labelled cells.
C: ST3GAL1 activity in HT1376sT and HT1376T cells was measured by using benzyl-T as acceptor substrate. Data are the mean ± SD of three experiments.
D: ST3GAL1 transcript abundance was measured by real time RT PCR and expressed as ST3GAL1 mRNA molecules/1000 β-actin molecules. Data are the mean ± SD of three experiments.
Fig. S1
