# Supplementary material for: Oxidative damage and response to Bacillus Calmette-Guérin in bladder cancer cells expressing sialyltransferase ST3GAL1
Source: BMC Cancer. 2018 Feb 17;18:198. doi: 10.1186/s12885-018-4107-1 (PMC5816560; doi:10.1186/s12885-018-4107-1)
Supplement: Supplementary file 2 — p values calculated with ANOVA, followed by Tukey multiple comparison test for data reported in Fig. 1 and Fig. 2 (PPTX 84 kb) [file 12885_2018_4107_MOESM2_ESM.pptx]

## Slide 1
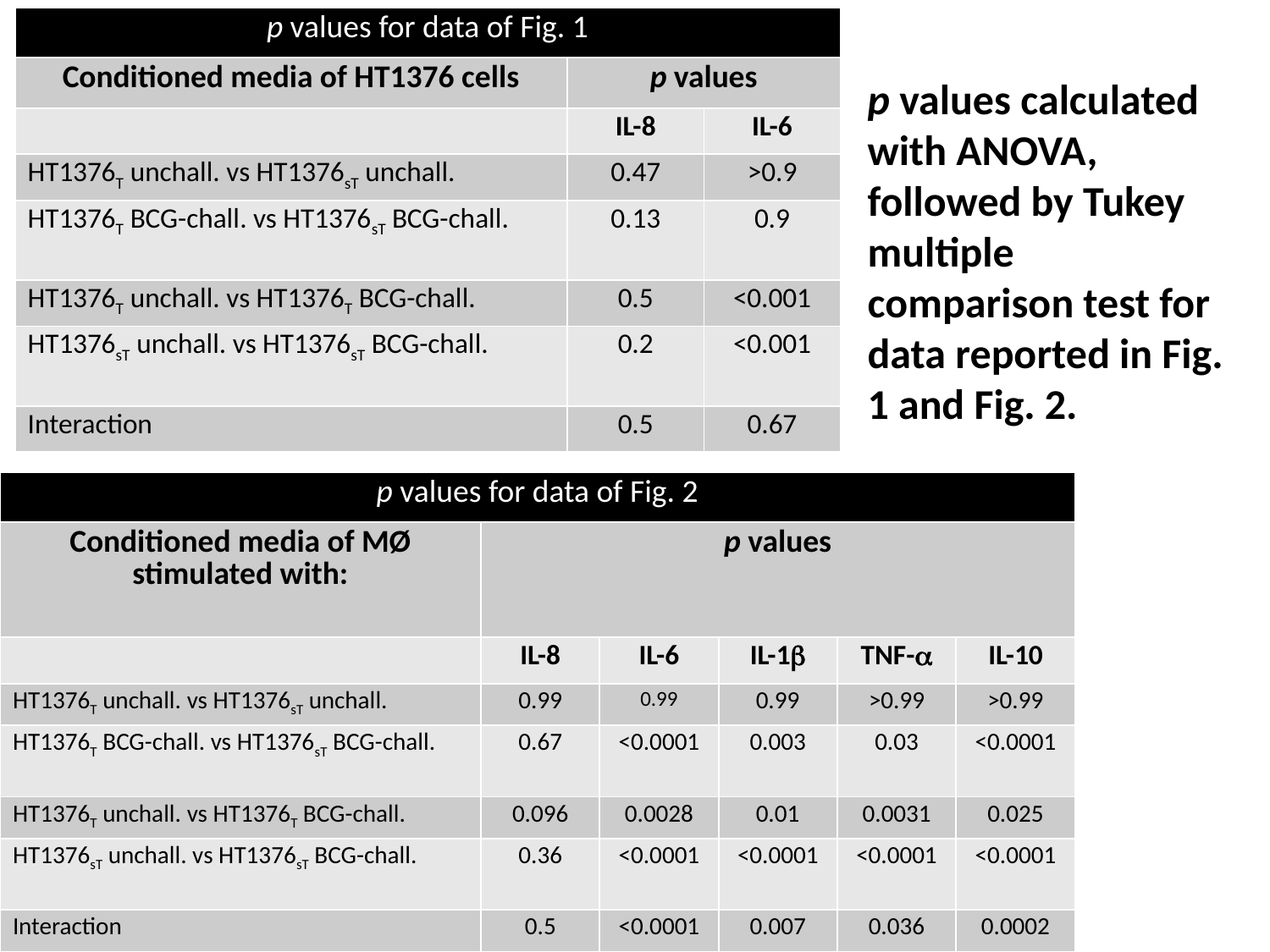

| p values for data of Fig. 1 | | |
| --- | --- | --- |
| Conditioned media of HT1376 cells | p values | |
| | IL-8 | IL-6 |
| HT1376T unchall. vs HT1376sT unchall. | 0.47 | >0.9 |
| HT1376T BCG-chall. vs HT1376sT BCG-chall. | 0.13 | 0.9 |
| HT1376T unchall. vs HT1376T BCG-chall. | 0.5 | <0.001 |
| HT1376sT unchall. vs HT1376sT BCG-chall. | 0.2 | <0.001 |
| Interaction | 0.5 | 0.67 |
p values calculated with ANOVA, followed by Tukey multiple comparison test for data reported in Fig. 1 and Fig. 2.
| p values for data of Fig. 2 | | | | | |
| --- | --- | --- | --- | --- | --- |
| Conditioned media of MØ stimulated with: | p values | | | | |
| | IL-8 | IL-6 | IL-1b | TNF-a | IL-10 |
| HT1376T unchall. vs HT1376sT unchall. | 0.99 | 0.99 | 0.99 | >0.99 | >0.99 |
| HT1376T BCG-chall. vs HT1376sT BCG-chall. | 0.67 | <0.0001 | 0.003 | 0.03 | <0.0001 |
| HT1376T unchall. vs HT1376T BCG-chall. | 0.096 | 0.0028 | 0.01 | 0.0031 | 0.025 |
| HT1376sT unchall. vs HT1376sT BCG-chall. | 0.36 | <0.0001 | <0.0001 | <0.0001 | <0.0001 |
| Interaction | 0.5 | <0.0001 | 0.007 | 0.036 | 0.0002 |
